# Self-Reported Anxiety and Depression in a Monocentric Cohort of Patients With Systemic Lupus Erythematosus: Analysis of Prevalence, Main Determinants, and Impact on Quality of Life

**DOI:** 10.3389/fmed.2022.859840

**Published:** 2022-03-29

**Authors:** Elena Elefante, Chiara Tani, Chiara Stagnaro, Viola Signorini, Beatrice Lenzi, Dina Zucchi, Francesca Trentin, Linda Carli, Francesco Ferro, Marta Mosca

**Affiliations:** Rheumatology Unit, Department of Clinical and Experimental Medicine, University of Pisa, Pisa, Italy

**Keywords:** lupus erythematosus systemic, anxiety, depression, patient reported outcomes, health related quality of life

## Abstract

**Aims of the study:**

To analyze the prevalence of self-reported anxiety and depression in a monocentric cohort of patients with Systemic Lupus Erythematosus (SLE); to study the main determinants and the impact on quality of life (QoL).

**Methods:**

A cross-sectional observational study including adult outpatients with SLE. Demographic and clinical data were analyzed: indices of disease activity (SELENA-SLEDAI); damage (SLICC-DI); comorbidities and concomitant therapies. The definitions for remission (DORIS) and “Lupus Low Disease Activity State” (LLDAS) were applied. At enrollment, each patient completed the following questionnaires: SF-36, FACIT-Fatigue, Lupus Impact Tracker (LIT), Systemic Lupus Activity Questionnaire (SLAQ), and the Hospital Anxiety and Depression Scale (HADS) in order to self-assess anxiety and depression symptoms. The Student *t*-test and Chi^2^ tests were conducted for univariate analysis. The Spearman test was used for linear correlation between continuous data. Multivariate analysis was performed by multiple linear and logistic regression.

**Results:**

One hundred fifty-four consecutive patients with SLE were enrolled, the majority female and Caucasian with a mean age = 43.3 ± 13.7 years. 79.9% were in LLDAS or remission. 36.4% had a SDI > 1. 13.7% of patients had concomitant fibromyalgia. 37.4% had symptoms indicating anxiety and 25% of depression according to the HADS questionnaire. In the multivariate analysis, patients with active disease were significantly more anxious and depressed (*p* < 0.01) compared to patients in LLDAS or remission. Fibromyalgia and older age were independently associated with anxiety and depression, respectively (*p* < 0.05). Active skin involvement was significantly linked to depression (*p* < 0.05). Higher scores on the HADS questionnaire (higher levels of anxiety and depression) were found to be significantly linked to patients’ perception of higher disease activity and worse quality of life, irrespective of disease activity, age and fibromyalgia.

**Conclusion:**

Symptoms of anxiety and depression are frequent in SLE patients, including outpatients with mild/moderate disease. Such symptoms have a significant negative impact on QoL and perception of disease activity, regardless of other factors. Moreover, disease activity, advanced age and fibromyalgia appear to be significantly linked to mood disorders. Assessing symptoms of the anxious-depressive spectrum in patients with SLE could lead to improvement in patients’ perception of health status and quality of life.

## Introduction

Systemic lupus erythematosus (SLE) is a chronic systemic autoimmune disease with fluctuating periods of exacerbation and remission. It typically affects young women and has a significant impact on daily living activities. Mortality in individuals with SLE has improved in recent decades (1) allowing the focus of care to shift toward improving patients’ Health-Related Quality of Life (HRQoL).

Several studies have shown that HRQoL of SLE patients is poorer than that of healthy controls (2). Of the various factors studied, mood disorders were found to be one of the most important determinants for poor HRQoL in patients with SLE (3, 4).

Physical and mental impairment caused by organ damage in SLE may seriously affect a patient’s ability to achieve life goals and work disability, leading to psychosocial stress (5). Moreover, reaction to a chronic illness, poor coping strategies, uncertainty about disease flare and prognosis may further predispose SLE patients to mood disorders (6).

Individuals with severe symptoms of anxiety and depression have reported difficulty in following their treatment plans resulting in failure to manage their illness (7).

Depression and anxiety can develop at different stages of SLE and can range in severity from mild symptoms to more severe disorders. A contradictory relationship exists between mood disorders and disease activity. Some studies have demonstrated that uncontrolled disease activity may lead to the development of depressive disorders, but “psychosocial factors” are considered the most frequent possible cause of depression in SLE. Although anxiety and depression represent a major cause of morbidity in SLE patients, there is little consensus on their prevalence. Generic diagnosis of depression is reported from 2 to 60% of patients, a diagnosis of major depression from 20 to 47% and a diagnosis of mood disorders ranges from 16.7 to 26.8%. Fatigue and weakness, which are often symptoms of SLE itself, are also reported as the most frequent depressive symptoms (88–90%) (8).

In light of this, early recognition of depression and anxiety in patients with SLE appears to be crucial for appropriate management: understanding the role of psychological factors may contribute to a more comprehensive understanding of SLE and its impact on a patient’s life. However, depression and anxiety are unfortunately often overlooked by non-psychiatric practitioners in clinical practice.

The aim of this study is therefore to analyze the prevalence of self-reported anxiety and depression in a monocentric cohort of patients with SLE, to study their main determinants and the impact of mood disorders on HRQoL.

## Materials and Methods

This is a cross-sectional, observational, monocentric study performed at the Rheumatology Unit, University of Pisa. Adult outpatients with a diagnosis of SLE who met the 1997 ACR or the 2012 SLICC classification criteria and who were monitored regularly at our Lupus clinic were included in the study between November 2020 and May 2021. The following data were collected for each patient at enrollment: epidemiologic and demographic characteristics; disease duration; cumulative organ involvement; comorbidities and concomitant treatments. Active disease manifestations were assessed at enrollment. In addition, disease activity was assessed using the Safety of Estrogens in Lupus Erythematosus National Assessment-Systemic Lupus Disease Activity Index (SELENA-SLEDAI) and the Physician Global Assessment (PGA); organ damage was evaluated by the SLICC-Damage Index (SDI). The DORIS definition of remission (*clinical*-SLEDAI = 0, PGA < 0.5 with stable maintenance antimalarials; low-dose corticosteroids -prednisone ≤5 mg/day- and/or maintenance immunosuppressives and/or biologics) (9) and the “Lupus Low Disease Activity State” (LLDAS) (SLEDAI ≤ 4, with no activity in major organ systems – renal, central nervous system (CNS), cardiopulmonary, vasculitis, fever, gastrointestinal, and no hemolytic anemia, no new active manifestations compared to the previous assessment, PGA ≤ 1, with stable prednisone ≤7.5 mg/day and standard maintenance doses of immunosuppressives and/or biologics) (10) were applied to the study cohort. At enrollment, each patient completed the following Patient Reported Outcomes (PROs): the Short Form-36 (SF-36) to assess HRQoL; the Functional Assessment Chronic Illness Therapy – Fatigue (FACIT) to assess fatigue; the Lupus Impact Tracker (LIT) to evaluate the impact of disease on patients’ daily living; and the Systemic Lupus Activity Questionnaire (SLAQ) for the self-assessment of disease activity.

The SF-36 addresses eight domains whose scores can be summarized into two global scores: the physical component summary (PCS) and the mental component summary (MCS). Each score ranges from 0 to 100, with higher values representing better self-perceived HRQoL.

The FACIT-F represents an example of a symptom-specific questionnaire. It assesses fatigue perceived in the physical, emotional, functional domains, its impact on daily activities and its social consequences, in a “recall period” of 7 days. It consists of 13 items and the score ranges from 0 to 52, with higher scores indicating a lower symptom incidence.

The LIT includes 10 questions about cognition, lupus medication, physical health, pain/fatigue impact, emotional health, body image, and planning/desires/goals. The LIT provides one summary score that captures the overall impact of lupus on patients’ health status. The score ranges from 0 to 100 and the lower the LIT score is, the less impact SLE is having on a patient’s life.

The SLAQ score includes a list of 24 symptoms. For each symptom, patients are asked to choose between four options: (a) mild; (b) moderate; (c) severe; (d) no symptoms. The items are weighted according to their clinical importance and grouped into 17 categories and each item can be scored from 0 to 3 according to symptom severity. The final SLAQ score can range from 0 to 47.

Finally, the Hospital Anxiety and Depression Scale (HADS) was used to self-assess symptoms of anxiety and depression symptoms (analyzed, for each symptom, both as a scale value and considering the cut-off of 8 points as a significant value). The HADS questionnaire is a 14-item instrument assessing symptoms of anxiety and depression. It consists of two subscales, HADS-D for depression and HADS-A for anxiety, each containing seven items from 0 to 3, with possible total scores each ranging from 0 to 21 for each subscale. A cut-off of 8 for either subscale indicates a positive screen for anxiety or depression. The questionnaire has a recall period of 7 days. HADS has been studied in several rheumatic diseases, including SLE (11–13).

The study was approved by the local Ethics Committee and patients were required to sign informed consent.

### Statistical Analysis

Continuous data are reported as median and interquartile range (IQR) or as mean and standard deviation (SD) as appropriate. Categorical data are reported as a percentage. The Student *t*-test and Chi^2^ tests were conducted for univariate analysis. The Spearman test was used for linear correlation between continuous data. Multivariate analysis was also performed by multiple linear and logistic regression for variables which were significantly associated within univariate analysis. All *p*-values less than 0.05 were considered statistically significant. Statistical analysis was performed using STATA 13 software.

## Results

One hundred fifty-four consecutive patients with SLE (1997 ACR or 2012 SLICC classification criteria) were included in the study. They were mainly female (92.8%) and of Caucasian ethnicity (94.8%), with a mean age at enrollment of 43.3 ± 13.7 years and a median disease duration of 10.5 years (IQR 6–21).

Table 1 summarizes the frequency of cumulative and active organ involvement of patients included in the study. At enrollment, the most frequent active disease manifestations were: cutaneous (16.2%), hematologic (14.9%), and articular (11.7%); 7.1% of patients had an active renal involvement. 21/154 patients (13.6%) had a SLEDAI > 4, with a median SLEDAI of 6 (IQR 6–8). At baseline, 123/154 patients (79.9%) were in LLDAS and of these, 103 (66.88% of the entire cohort) also satisfied the DORIS definition of remission (on or off treatment).

**TABLE 1:** Cumulative and active organ involvement of enrolled patients.

Type of organ involvement	Cumulative (% of patients)	Active (% of patients)
Articular	74.51%	11.69%
Hematologic	60.13%	14.94%
Skin	57.52%	16.23%
Renal	39.22%	7.14%
Serositic	11.76%	2.6%
Neuropsychiatric SLE	7.95%	1.34%

56/154 (36.4%) of patients had at least one organ damage (SDI better than SLICC > 1), with a median SDI of 1 (IQR 1–2). 21/154 (13.7%) patients had a diagnosis of concomitant fibromyalgia (FM).

In terms of ongoing treatment, most patients were taking Hydroxychloroquine (85.71%) and low-dose steroids (55.19%) with a median daily dose of 2 mg (IQR 0–4) of 6-methylprednisolone. 41.56% of the cohort was on immunosuppressants and 17.16% was on treatment with biologic DMARDs, mainly Belimumab.

### Analysis of Patient Reported Outcomes

**FIGURE 1:**
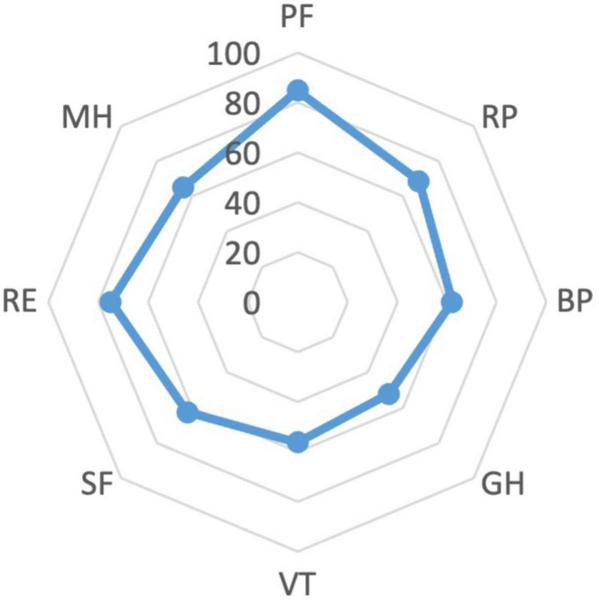
Scores of the single items of SF-36.

The cohort’s median scores of the PCS and the MCS of the SF-36 were 48.44 (IQR 41.91–54.27) and 44.04 (IQR 38.02–52.45), respectively. The lowest scores related to the items of General Health (GH) (52, IQR 37–57) and Vitality (VT) (56.25, IQR 37.5–68.75). The scores for the SF-36 items are reported in Figure 1.

The cohort’s median score of the FACIT questionnaire was 40 (IQR 32–46) and the median score of the LIT was 27.5 (IQR 7.5–42.5), suggesting a low/moderate impact of disease on patients’ daily living. Finally, the SLAQ questionnaire for the self-evaluation of disease activity obtained a median score of 10 (IQR 5–15).

37.4% of the entire patient cohort had symptoms indicating anxiety, while 25% of the cohort had symptoms indicating depression according to the HADS questionnaire (HADS-A scores for anxiety and HADS-D for depression ≥8); the median HADS score for anxiety was 7 (IQR 3–10) and for depression was 5 (IQR 2–8).

### Analysis of the Major Determinants of Mood Disorders

In the univariate analysis, we evaluated the correlation between symptoms of anxiety and depression and the demographic and clinical characteristics of patients enrolled.

We found a significant positive correlation between age at enrollment and symptoms of anxiety and depression (*p* < 0.05), while no correlation emerged in terms of sex and disease duration. As for disease activity, we found that anxiety and depression were significantly more frequent among patients not in LLDAS compared to patients in LLDAS or remission (*p* < 0.01), whereas no correlation was found between the HADS and the SELENA-SLEDAI scores. We did not observe a significant difference between patients in LLDAS and patients in remission, either for anxiety scores (*p* = 0.8) or for depression scores (*p* = 0.78).

On the other hand, in the univariate analysis the SDI score was significantly associated with both anxiety and depression (*p* < 0.05).

As for single active organ involvement, we only observed an association between active skin involvement and depressive symptoms (*p* < 0.05). No other active disease manifestations appeared to influence mood disorders in our cohort. In addition, no significant association emerged between HADS results and ongoing treatment. In particular, there was no significant correlation between ongoing glucocorticoids and their daily dose and the existence of anxiety (*p* = 0.4) or depression (*p* = 0.3).

Finally, as expected, patients with fibromyalgia were significantly more anxious and depressed (*p* < 0.05) compared to patients who did not suffer from this.

In the multivariate analysis, fibromyalgia proved to be an independent factor linked to anxiety (OR = 2.99, *p* < 0.05), while older age resulted independently linked to depression at the limits of significance (OR = 1.03, *p* = 0.05). On the other hand, the correlation between HADS score, both for anxiety and depression, and organ damage (SDI) was lost when adjusting for age at enrollment (*p* = 0.61 for depression and *p* = 0.21 for anxiety). Active skin involvement also lost its correlation with depression when adjusting for the other factors considered (*p* = 0.4). Moreover, we confirmed that patients with active disease were significantly more anxious (OR = 0.24, *p* < 0.01) and more depressed (OR = 0.30, *p* < 0.01) than patients in LLDAS or remission, irrespective of age at enrollment and fibromyalgia accordingly. Table 2 summarizes the results of the multivariate analysis.

**TABLE 2:** Determinants of mood disorders: Results of the multivariate analysis.

	LLDAS	FM	Age at enrollment	SLICC/DI
ANXIETY	OR = 0.24	OR = 2.99	OR = 1.02	OR = 1.21
	*p* < 0.01**	*p* < 0.05*	*p* = 0.22^§§^	*p* = 0.21*
DEPRESSION	OR = 0.30	OR = 2.4	OR = 1.03	OR = 1.08
	*p* < 0.01*	*p* = 0.09*	***p* = 0.05^§^**	*p* = 0.61*

*After adjusting for age at enrollment.

**After adjusting for age at enrollment and fibromyalgia.

*^§^After adjusting for LLDAS, SLICC-DI, fibromyalgia, and active skin involvement.*

*^§§^After adjusting for LLDAS, SLICC-DI, and fibromyalgia.*

*Bold values are statistically significant values.*

### Analysis of the Correlation Between Mood Disorders and Patient Reported Outcomes

Finally, we analyzed the link between mood disorders and the other PROs in our cohort.

Using the Spearman test, we observed a very strong correlation between the HADS score (scale value) and the scores of all other PROs administered (SF-36, FACIT, LIT, and SLAQ), with the strongest link evident in the mental component of SF-36 as reported in Table 3.

**TABLE 3:** Correlation between HADS and other PROs.

	Anxiety (HADS-A score)	Depression (HADS-D score)
FACIT	*p* < 0.0001	*p* < 0.0001
	*r* = −0.54	*r* = −0.62
LIT	*p* < 0.0001	*p* < 0.0001
	*r* = 0.66	*r* = 0.66
SF-36 (PCS)	*p* < 0.0001	*p* < 0.0001
	*r* = −0.45	*r* = −0.48
SF-36 (MCS)	*p* < 0.0001	*p* < 0.0001
	*r* = −0.79	*r* = −0.76
SLAQ	*p* < 0.0001	*p* < 0.0001
	*r* = 0.55	*r* = 0.57

In the multivariate analysis, after adjusting for disease activity, age and fibromyalgia, higher scores on the HADS questionnaire (suggestive of greater anxiety and depression) were found to be significantly linked to: patients’ perception of higher disease activity (SLAQ, *p* < 0.001); a greater burden of disease on patients’ life (LIT, *p* < 0.001); higher levels of fatigue (FACIT, *p* < 0.001) and worse HRQoL (PCS, *p* < 0.01; MCS, *p* < 0.001) (Table 4).

**TABLE 4:** Correlation between HADS score and the other PROs: Results of the multivariate analysis.

	FACIT	LIT	PCS	MCS	SLAQ
ANXIETY*	*p* < 0.001	*p* < 0.001	*p* < 0.01	*p* < 0.001	*p* < 0.001
	Coef. −0.19	Coef. 0.12	Coef. −0.12	Coef. −0.31	Coef. 0.33
DEPRESSION**	*p* < 0.001	*p* < 0.001	*p* < 0.001	*p* < 0.001	*p* < 0.001
	Coef. −0.2	Coef. 0.1	Coef. −0.12	Coef. −0.25	Coef. 0.3

*After adjusting for age at enrollment, fibromyalgia and LLDAS.

**After adjusting for age at enrollment and LLDAS.

## Discussion

This cross-sectional study investigated the prevalence and main determinants of mood disorders in a monocentric cohort of patients with SLE. We also wanted to explore the impact of mood disorders on patients’ HRQoL and illness perception. We enrolled consecutive SLE outpatients regularly monitored at the Rheumatology Unit of Pisa. Almost 80% of patients enrolled were in LLDAS or remission and the most frequent active disease manifestations at enrollment were cutaneous, hematologic and articular. Particularly, no patients had active neuropsychiatric SLE manifestations.

Using the self-administered HADS questionnaire, we found that 37.4% of patients had symptoms indicating anxiety and 25% of patients had symptoms indicating depression, confirming that mood disorders are quite frequent among patients with SLE, even in an outpatient cohort reporting an overall well-controlled disease.

Data from current literature show a high heterogeneity of prevalence values of mood disorders among SLE patients. This heterogeneity may be due to a lack of standardized definitions and instruments used to measure mood disorders and to a variability in demographic characteristics of patients included. According to a recent meta-analysis including 14 studies that used the HADS-D (for a total of 1,238 SLE patients) and 12 studies that used the HADS-A (for a total of 1,099 SLE patients), the pooled prevalence of depression was 35.0% while that of anxiety was 25.8% (14). An even higher prevalence of mood disorders was found in a study by Bachen et al. as follows: of 326 Caucasian women with SLE, 47% had a lifetime diagnosis of major depressive disorder and 49% had a lifetime diagnosis of anxiety disorder (15).

In our cohort, mood disorders appeared to be independently associated with disease activity, along with advanced age and concomitant fibromyalgia. Although our study did not find any correlation between the SELENA-SLEDAI score and the HADS, we observed that patients in LLDAS or remission were significantly less anxious and depressed compared to active patients. Tay et al. demonstrated that anxiety (40.9 vs. 21.8%, *p* = 0.002) and depression (15.5 vs. 6.4%, *p* = 0.025) were significantly more frequent among lupus patients compared to healthy controls. In particular, they observed that active disease was associated with more severe anxiety regardless of the presence or absence of concomitant depression (16).

In terms of active disease manifestations, we found that skin lesions were significantly linked to depression in our cohort. It is well-known from the literature that patients’ main concerns are often related to mild manifestations, such as musculoskeletal and mucocutaneous ones, due to their greater impact on patients’ degree of functioning and body image, rather than to major organ involvement and this may determine a discordance between patient and physician assessment of SLE disease status (17, 18). Similarly to the results of this study, Eldeiry et al. recently observed a prevalence of anxiety and depression of 34% and 27%, respectively, in a cohort of 341 SLE patients. Their study observed that patients with skin involvement were at higher risk of having coexisting anxiety and depression (19). Fischin et al. demonstrated in a cross-sectional study of the LuLa cohort that scarring changes of the skin are some of the factors which are significantly linked to catastrophizing in SLE patients (20). Moreover, patients with primary Cutaneous Lupus Erythematosus (CLE) also seem to have poorer HRQoL compared to the general population and to patients with other dermatological and medical conditions. Emotions, daily functioning, general and mental health are specific areas which are significantly affected in CLE patients (21, 22).

In our cohort, 13.7% of patients had concomitant fibromyalgia and proved more likely to be anxious than patients who did not suffer from this, irrespective of other factors considered. Such findings are in line with other studies. According to a cross-sectional data analysis from the RELESSER-Transversal Spanish Registry, for example, SLE patients with FM showed higher prevalence of depression compared to non-FM-SLE patients (53.1 vs. 14.6%, *p* < 0.001) (23).

It is also significant to note that this study also observed that patients with self-reported symptoms of anxiety and depression reported a worse HRQoL, greater levels of fatigue and a higher burden of disease on their daily living, irrespective of confounding factors. In fact, we found a strong negative correlation in our cohort between the HADS score and the SF-36, particularly in terms of items relating to mental health MCS (*r* = −0.79 for anxiety and *r* = −0.76 for depression) suggesting that patients with mood disorders had a worse quality of life. We also found a strong negative correlation between the HADS and the FACIT scores (*r* = −0.54 for anxiety and *r* = −0.62 for depression), indicating that patients with anxious and depressive symptoms independently reported a higher level of fatigue. Finally, we found a significant positive correlation between the HADS and the LIT scores (*r* = 0.66, both for anxiety and depression) suggesting that patients reporting symptoms of anxiety and depression perceive a greater burden of disease on their daily life regardless of disease activity, fibromyalgia and other factors considered.

These data generally confirm what has been demonstrated in prior research on HRQoL in SLE. Doria et al. already demonstrated that anxiety, depression and joint pain were the major determinants of HRQoL impairment and organ damage influenced HRQoL mostly through depression in an Italian cohort of SLE patients (24). In a Southern California SLE cohort, depression was the main factor correlated with lower scores in all SF-36 domains, while the SLEDAI had no such correlation (25).

More recently, Mok et al. demonstrated that both the MCS and PCS of SF-36 score was significantly linked to HADS depression (*p* < 0.001) and anxiety score (*p* < 0.001), after adjustment for confounding factors. They also found that depression may aggravate somatic symptoms such as muscle and body aches, fatigue and the need for medical attention (26).

Over the last year, a cross-sectional analysis was performed on adult SLE patients of the California Lupus Epidemiology Study (CLUES), a prospective longitudinal cohort, demonstrating that major depression was associated with significantly worse HRQoL, evaluated with 12 PROMIS domains. Interestingly, this link was also confirmed in areas of particular relevance to SLE, such as Fatigue and Satisfaction in Social Roles, after adjustment for age, sex, race/ethnicity, SLEDAI score, SDI score, BMI, and household income (27).

Finally, it is worth underlining that we found a strong positive correlation in the Spearman test between the HADS score and the SLAQ score (*r* = 0.55 for anxiety and *r* = 0.55 for depression), which expresses how patients evaluate their disease activity. This positive correlation between the HADS and the SLAQ score suggests that mood disorders may influence illness perception of patients with SLE. In a recent study, a lack of association was observed between disease activity and organ damage with illness perception which conversely resulted to be linked to depression, anxiety, the severity of fatigue and pain as well as sleep quality. Therefore, objective factors may be less important when it comes to illness perception and early psychotherapeutic intervention may protect against negative disease perception in the later stages of a chronic disease such as SLE (28). In a previous study, we investigated the role of alexithymia and depression on negative perceptions of illness in a group of 100 consecutive SLE outpatients attending our clinic. We found that patients who experienced greater depressed mood perceived their disease as chronic with worse consequences on their life and lower treatment efficacy, were less able to understand the disease, and displayed stronger emotional responses to the illness (29).

We acknowledge that this study presents certain limitations, namely the fact that we have not considered certain factors such as education level and socio-economic status, which may influence HRQoL and mood disorders. Moreover, patients with HADS suggestive for anxiety and depression were not evaluated by a specialist for a definite psychiatric diagnosis. Finally, we have included outpatients in our study and this may have limited the number of patients with severe active disease manifestations. Despite such limitations, we believe that our study has certain strengths. First of all, the large number of patients enrolled, all of which were regularly monitored at the same center, and thus fully characterized from a clinical point of view. Moreover, we have explored the correlation of mood disorders with both generic and SLE-specific PROs, which give complementary information on patient health status. In particular, we believe that the generic questionnaire SF-36 explores many domains of patients’ life and allows the comparison with different cohorts of patients; on the other hand, the LIT questionnaire, which is a Lupus-specific tool, is able to highlight some aspects of patients’ quality of life linked to peculiar SLE-related factors.

## Conclusion

Mood disorders appear to be frequent in SLE patients, even among outpatients with mild to moderate disease, and determine a strong negative impact on quality of life and perception of disease activity, regardless of other factors. Our results therefore indicate that rheumatologists should routinely assess the presence of psychological problems in their SLE patients. In the context of a multidisciplinary management, collaboration with expert clinical psychologists for targeted interventions on symptoms of depression or anxiety may be essential to the provision of appropriate treatment options with the final aim of improving patients’ perception of health status and quality of life.

## Data Availability Statement

The raw data supporting the conclusions of this article will be made available by the authors, without undue reservation.

## Ethics Statement

The studies involving human participants were reviewed and approved by the Comitato Etico Area Vasta Nord Ovest. The patients/participants provided their written informed consent to participate in this study.

## Author Contributions

EE contributed to the acquisition of data and analysis and interpretation of data, and was in charge of writing the manuscript. CT and MM contributed to the conception and design of the study, acquisition of data, and writing of the manuscript, and was responsible for the analysis and interpretation of data. CS, VS, BL, DZ, FT, LC, and FF contributed to the acquisition of data and drafting the manuscript. All authors read and approved the final version of the article.

## Conflict of Interest

The authors declare that the research was conducted in the absence of any commercial or financial relationships that could be construed as a potential conflict of interest.

## Publisher’s Note

All claims expressed in this article are solely those of the authors and do not necessarily represent those of their affiliated organizations, or those of the publisher, the editors and the reviewers. Any product that may be evaluated in this article, or claim that may be made by its manufacturer, is not guaranteed or endorsed by the publisher.
